# Genome-wide identification of the regulatory targets of a transcription factor using biochemical characterization and computational genomic analysis

**DOI:** 10.1186/1471-2105-6-275

**Published:** 2005-11-18

**Authors:** Emmitt R Jolly, Chen-Shan Chin, Ira Herskowitz, Hao Li

**Affiliations:** 1 Department of Biochemistry and Biophysics, University of California, San Francisco, 1700 4^th ^Street, San Francisco, CA 94143, USA

## Abstract

**Background:**

A major challenge in computational genomics is the development of methodologies that allow accurate genome-wide prediction of the regulatory targets of a transcription factor. We present a method for target identification that combines experimental characterization of binding requirements with computational genomic analysis.

**Results:**

Our method identified potential target genes of the transcription factor Ndt80, a key transcriptional regulator involved in yeast sporulation, using the combined information of binding affinity, positional distribution, and conservation of the binding sites across multiple species. We have also developed a mathematical approach to compute the false positive rate and the total number of targets in the genome based on the multiple selection criteria.

**Conclusion:**

We have shown that combining biochemical characterization and computational genomic analysis leads to accurate identification of the genome-wide targets of a transcription factor. The method can be extended to other transcription factors and can complement other genomic approaches to transcriptional regulation.

## Background

The availability of genome sequences for multiple species and large-scale gene expression data has led to the development of computational genomic approaches to transcriptional regulation. A challenge in the field is the accurate genome-wide identification of the regulatory targets of transcription factors (TF), which is a necessary step towards reconstructing cellular transcriptional networks. A number of functional genomic approaches have been developed to tackle this problem. For example, ChIP-chip (chromatin immunoprecipitation followed by hybridization to DNA chip) technology has been applied on a large scale to map the location of transcription factors in the yeast genome [1,2]. Gene expression profiling of cells in which a transcription factor is either overexpressed or deleted has also been used to identify targets [3-8]. While these are powerful approaches to systematically identifying target genes, they also have limitations. For example, ChIP-chip experiments performed under a specific condition may not identify the correct targets of a factor if that factor is not activated, or may only identify a subset of the target genes if the factor works with other TFs in a combinatorial fashion, and searching for all conditions under which a factor may be functional is a daunting task. In addition, physical binding to a promoter as detected by a ChIP-chip experiment may not necessarily imply regulation. Similarly, the response of the genome to transcription factor perturbation may be condition dependent and may involve a transcriptional cascade with many indirect targets. Together with noise in the experimental measurements, these limitations also make it difficult to estimate the false positive and false negative rates.

Predicting the binding site and target genes of a TF based on sequence analysis is an approach complementary to functional analysis. The most straightforward approach, predicting the targets of a TF based on consensus binding site lacks the desired sensitivity and specificity, often identifying many more targets than are believed to be biologically relevant. Using a position specific weight matrix, typically derived from the probability of occurrence of 4 nucleotides at each single base position based on known examples of binding sites is better than using a consensus sequence as it allows a quantitative measure of the strength of the binding site. However, this procedure still produces a large number of false positives under conditions that achieve reasonable sensitivity.

We present a method for target prediction that does not depend on gene expression or other functional genomics data. The method selects target genes by combining the follow criteria: 1) quantitative binding affinity of the potential binding site based on biochemical characterization of the factor; 2) position of the site relative to the gene start; 3) conservation of the site in sequence and position across multiple closely related species. These criteria have been used individually before to predict binding sites or target genes. For example, binding affinity data from in vitro experiments has been used to scan potential target genes in a genome [9,10] and conservation across species has been explored to identify regulatory elements [11-16]. Location preference of true binding sites has also been observed before [12,17-19]. Here we show that these criteria can be combined to generate target predictions with high sensitivity and specificity. In addition, a mathematical analysis of the multiple selection criteria allows us to accurately estimate the false positive rate and the total number of targets in the genome. Such a calculation is not possible if targets are selected by a single criterion.

We start by characterizing the binding site sequence requirements for the TF using biochemical and genetic experiments, and use the data to quantitatively measure the binding affinity of potential binding sites. We then analyze the positional distribution of the true binding sites computationally; using a carefully selected true target set, and test the relevance of location directly by *in vivo *experiments. Finally, we filter for genes with binding sites conserved across multiple species. Combining conservation with affinity and position, we predict a set of target genes and calculate false positive and false negative rates. We demonstrate this approach by predicting the regulatory targets of Ndt80, a key transcription factor that directs expression of middle sporulation genes in yeast. We choose Ndt80 as it exemplified the difficulty of predicting target genes with high specificity. It is known that most genes in the genome with the consensus Ndt80 binding site are not regulated by Ndt80 [20].

Sporulation is initiated in diploid **a**/α cells in response to starvation and is characterized by the sequential expression of early, middle, mid-late and late genes [20,21]. Genome-wide expression profiling [20,22] has revealed that ~500 genes are induced during sporulation, of which about one third were classified as meiotic middle genes based on their expression profiles [20]. Most meiotic middle genes, necessary for exit from meiotic prophase and for meiotic nuclear division and spore morphogenesis [20,23] are regulated by Ndt80, which binds to the middle sporulation element (MSE), defined as gNCRCAAAW [24,25]. The yeast genome contains the Ndt80 core consensus binding site, CRCAAA, upstream of about 2000 genes. Using our method, we identified 115 genes as putative Ndt80 targets, among these ~40 are predicted to be false positives. We estimated that the total number of Ndt80 targets in the genome is ~170.

## Results

### Ndt80 binding sequence requirements *in vitro *

**Figure 1:**
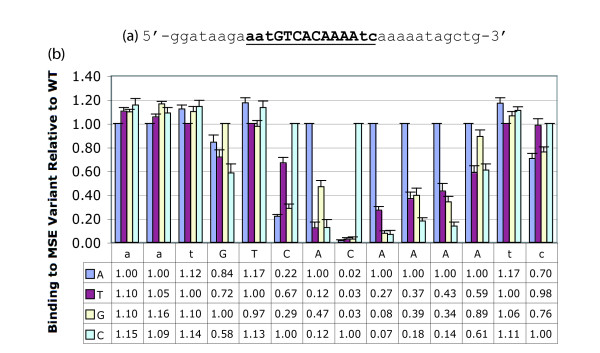
Ndt80 binds to MSE Variants Differently *in vitro*. Purified MBP-ndt80, 51–350 was incubated with 35 base-pair radio-labeled oligos containing a centralized MSE (underlined capital letters) (a) or single base MSE variants and analyzed by EMSA (b). Single base-pair variants three nucleotides 5' and two nucleotides 3' of the central MSE sequence were also tested. All EMSA bands were quantitated relative to the wild-type MSE where the percent of labeled wild-type MSE shifted by Ndt80p was designated 1. The same data was shown both in the graph and in the table.

We determined the binding preferences of the Ndt80 DNA binding domain (aa 51–350) fused N-terminally to Maltose binding protein (MBP-Ndt80 51–350) for the *SPO77 *MSE using EMSA analysis [26-28]. The *SPO77 *MSE (located at -152 from the start of translation) was chosen because it has good homology to the Ndt80 consensus [22,24,25] and because a single copy was sufficient to direct transcription [20]. We systematically examined the effects of every alteration at each position of a 14 bp region, which consisted of the 9 bp core sequence GTCACAAAA together with three upstream and two downstream base-pairs, presented as the central region of a 35 bp oligonucleotide (Figure 1a). The quantitative data from the mutational analysis is shown in Figure 1b. This study complements two previous binding preference studies, performed while this work was in progress [26,29].

We confirmed that most binding determinants are in the 9 bp core MSE. Of the five positions systematically mutated outside the core, only changes to the second position downstream of the MSE (+11) had any effect on binding and these were minor (Figure 1b). Within the core MSE, C5 is essential, as a change to any other nucleotide essentially abolished binding. The A4 and A6 positions are next most important, exhibiting significant decreases in binding with any change and severe restriction of binding with two of the three changes. The C3, A7 and A8 positions are also significant. For those positions where similar changes have been examined, the data of this study and that of Pierce et al are in excellent agreement [29]. These two studies generally agree with the more restricted data set of Lamoureaux et al [26], except for their conclusion that at C5, only a change to G severely restricts activity.

### Ndt80 binding sequence requirements *in vivo *

**Figure 2:**
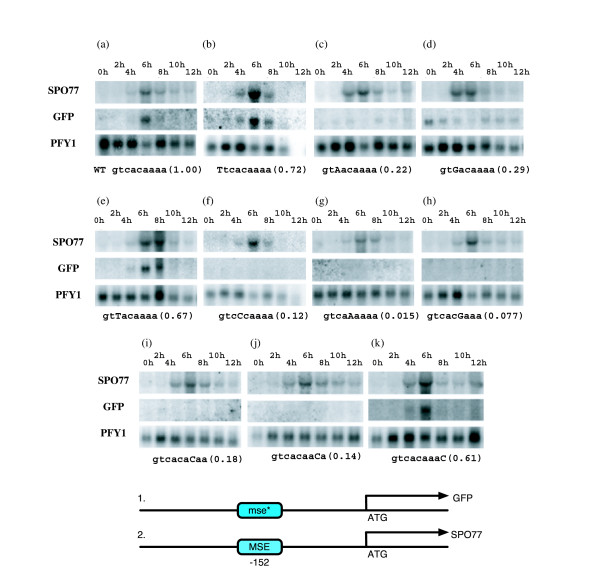
MSE sequence variations *in vivo *affect RNA levels through the *SPO77 *promoter. Shown at the bottom is a schematic of the heterologous *in vivo *chromosome constructs at the *SPO77 *locus. At one *SPO77 *locus, we replaced *SPO77 *with *GFP*, and the wild type MSE with mse*, which differs from the wild type by a single base mutation, written in capital letters (Except for (a), where the mse* is the same as the wild type.). The second *SPO77 *locus is unaltered. The resulting diploid SK1 strains were sporulated for 24 hours in liquid. Whole genomic RNA was extracted from these cells at 0,2,4,6,8,10, and 12 hours of sporulation, run on a denaturing polyacrylamide gel, transferred to a nitrocellulose membrane and probed sequentially with ^32^P labeled probes to *SPO77*, *GFP *and *PFY1*. Included in the parenthesis is the *in vitro *binding of Ndt80 to each MSE variant relative to the wild type.

A subset of the mutations were analyzed *in vivo *during sporulation, using a strain having one authentic *SPO77 *locus, so that sporulation was unimpaired, and a second *SPO77 *locus encoding GFP and driven either by the wide type promoter or one having an altered MSE (Figure 2). Quantitative analysis of the *in vivo *expression is included in the supplementary material (see Additional files 1 and 2). By comparing the RNA levels from the GFP construct with that from *SPO77 *expressed in the same cells and normalizing both to expression of an internal control, we were able to make an accurate determination of the effect of the MSE mutations on expression. When driven by the wild type MSE, *GFP *was induced at levels similar to *SPO77 *RNA with similar timing (Figures 2a), indicating that this system could be used to test expression of GFP driven by mutant MSEs.

With the exception of position 2, where nucleotide identity did not affect binding (Figure 1), we tested the effects of at least one nucleotide change at each MSE position on expression during sporulation (Figures 2). Overall, we find excellent agreement between our *in vivo *expression measurements and the *in vitro *binding data (Compare Figures 1 and 2). Importantly, these data validate our finding *in vitro *that changes to the C5 position other than G are very deleterious, as a C5A change has essentially no activity *in vivo *(Figures 2g). Additionally, by testing all changes at position C3 (Figures 2c–e), we could show that even small changes in *in vitro *binding are significant for expression *in vivo*.

### The location of the MSE is important for sporulation specific gene induction *in vivo *

Although total genomic MSEs are found in widely varying positions [22,24,25], our computational analysis of positive targets showed that functional MSEs are distributed in a preferred window between -75 to -300 bps upstream (see next section) of the translation start point. We therefore tested positional dependence of MSE activity by changing the endogenous MSE at -152 to a nonfunctional sequence (gtcaAaaaa) and positioning a consensus MSE at either-450 or -50. These MSEs mediate very little, if any *GFP *expression. Lack of gene expression is not due to inhibition of RNA synthesis, as normal *GFP *RNA synthesis is observed when the positionally altered variants also have the normal MSE at position -152 (Additional file 3). We conclude that MSEs position is important for function.

### Identification of the test set of middle genes utilized in computational analysis

To get a highly specific set of target genes as our test set, we define middle genes as those exhibiting less than a 1.2 fold change at 2 hours and greater than 3 fold change at 5 hours following the initiation of sporulation. Using these criteria, we identified 68 middle sporulation ORFs from the microarray data published by Chu et al.[20]. Of these 68 ORFs, 54 have multi-species sequence data (see method) and the Ndt80 core consensus binding site CRCAAA. These genes were used as our test set. 36 genes in this test set overlap with 62 strongly induced middle sporulation genes independently identified by Primig et al. via clustering analysis [21].

### The location of the MSE is specifically distributed in middle sporulation genes

**Figure 3:**
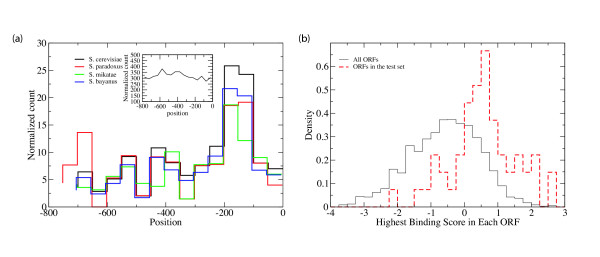
(a) Functional MSEs are located in preferred positional windows. Genes in the test set were analyzed for the location of the core MSE sequence, CRCAAA, in four yeast species. The inset shows the distribution of the locations of the MSE in the whole genome promoters. All the distributions were normalized by the number of available promoter sequences at the given location. (b) The distributions of binding scores for the MSE sites in the test gene set (red dashed curve) and in the whole genome (solid black curve).

We used a statistical analysis that compares the location of MSE relative to the translation start site in the test set with that in the whole genome promoters to determine whether there is a positional preference for MSE in the true target sets. We find that MSEs are highly localized between positions -75 to -300 from the translation start site in the test set (Fig 3a). In striking contrast, the MSE sequences in the whole genome promoters do not have a preferential location (Fig 3a, inset), suggesting that MSE location might be a determinant of middle gene activity. To pursue this issue further, we asked whether positional bias of the core MSE sequence, CRCAAA, of the test set promoters is conserved between *S. cerevisiae *and the orthologous promoters in *S. bayanus*, *S. mikatae*, and *S. paradoxus *[11,30]. We found that the positional distribution of the CRCAAA motif in all the other three species have similar preference (Fig. 3a), whereas no preference is observed for MSE sequences in the whole genome promoters.

### Distribution of the binding affinity of potential Ndt80 binding sites in the genome

There are over two thousand genes in the *S. cerevisiae *genome whose promoter (800 base pairs upstream) contains the Ndt80 core consensus binding motif CRCAAA. Given the modest number of middle genes, it is likely that most of these matches are false positives. It might be possible to discriminate against the false positives using quantitative biochemical data from the *in vitro *binding assay, since bases matching the degenerate symbols in the core motif and/or from the flanking sequences can contribute to the binding affinity (Figure 1). We therefore extracted the N5-CRCAAA-N3 motifs in all *S. cerevisiae *promoter sequences and scored each motif using the *in vitro *binding data, which we converted to binding affinity relative to the wild type *SPO77 *motif (see Materials and Methods). The resulting relative binding affinity reflects the contributions from the degenerate core position (R) and the flanking sequences. We observe that the distribution of relative binding affinities for matches in the test middle sporulation gene set is distinct from the set of all the matches in the genome (presumably mostly false positives) (Figure 3b). Whereas the mean of the genome-wide distribution is -0.58, with a standard deviation of 1.05, that of the reference set shifted by about one standard deviation towards a higher score, showing a mean of *m*_*s *_= 0.57 and a standard deviation of σ_*s *_= 0.96. These data show that the false positive matches can be discriminated to certain degree based on the quantitative binding affinity. For example, using *m*_*s *_- σ_*s *_as a cutoff score and assuming Gaussian distribution, we would eliminate about 50% false positives, while keeping more than 80% of true positives.

### Predicting the direct targets of Ndt80 in the genome

**Figure 4:**
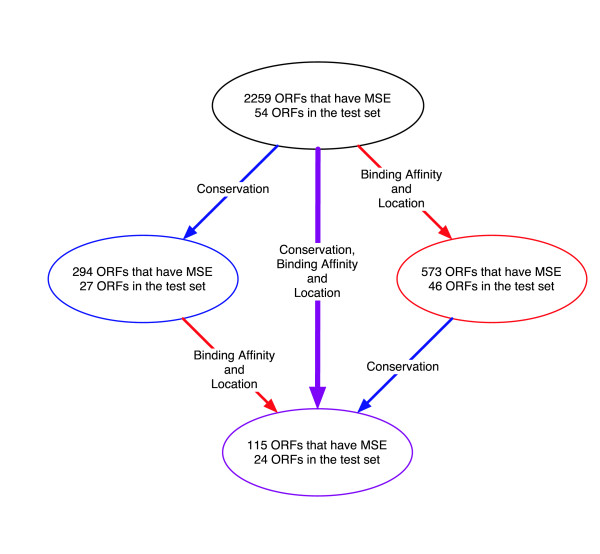
Schematic diagram of the effect of selection by the two different filters on two gene sets- the candidate Ndt80 targets (2259 ORFs) and the test set (54 ORFs). One filter is the binding score and location, and the other filter is conservation across 4 species. The number of ORFs in each set passing the first, the second, and both filters are indicated in the ovals. These numbers were used to estimate the false positive and false negative rates of the target prediction based on a simple model (see method for details).

We have shown that the potential Ndt80 binding sites in the promoters of middle genes differ from their non-middle gene counterparts by having on average a higher binding affinity and positional preference. With the availability of complete sequences of several yeast species, it is possible to use evolutionary conservation as an additional filter to select true binding sites, since functional sites are more conserved due to selection pressure. We therefore searched for the direct targets of Ndt80 using the following criteria: 1) the selected ORF should have at least one core motif N5-CRCAAA-N3 with binding affinity score higher than a specified cutoff (*m*_*s *_- σ_*s *_); 2) the motif is located between -80 to -400 from the translation start site; and 3) the core motif is conserved in a number of yeast species (see methods for details). To assess the predictive value of this selection scheme, we compared its performance on the middle gene test set with that on the 2259 ORFs whose promoter has at least one core MSE motif. When we use conditions 1, 2 and demand conservation of the core motifs in all 4 species, we identify 115 ORFs in *S. cerevisiae *as potential Ndt80 regulatory targets (Figure 4). These combined criteria reduced the total number of predictions by 20 fold, and thus greatly reduced the number of false positives. On the other hand, the predicted ORFs still include 44% of the ORFs in the test set having the MSE motif (24 out of 54). This demonstrates that our procedure can successfully discriminate between bonafide middle genes and false positives. Furthermore, the computational analysis also predicted 91 new genes as potential Ndt80 targets.

### Calculating the false positive and the false negative rate

To gauge the accuracy of the prediction, it is important to ask how many of the predicted genes are false positives and how many true targets are missed. In a functional genomics approach, the false positive rate is typically estimated by experimental verification of the predicted targets. Here we develop a mathematical approach that allows us to estimate both false positive and false negative rate, and consequently the total number of targets in the genome. Crucial to this approach are: 1) a set of true targets known with high confidence; and 2) multiple independent filters that can be used to select potential targets. The idea is illustrated in Figure 4 where we start with a high confidence positive set of 54 genes and 2259 potential targets with the consensus MSE site in their promoters. We believe that the 54 genes are the authentic targets of Ndt80 as they passed the stringent criterion of gene expression (exhibiting less than 1.2 fold change at 2 hours and greater than 3 fold change at 5 hours following the initiation of sporulation) and also posses a consensus MSE in their promoters. We now apply two different filters individually and jointly. One filter selects genes whose binding sites score is larger than (*m*_*s *_- σ_*s *_) and the motif is located between -80 to -400 from the translation start site (condition 1 and 2 of the selection in the previous section). The other filter selects genes whose promoter have the MSE site conserved across 4 species (condition 3). The fractions of true positives passing the two filters can be estimated from the test set. There are three unknowns: the fractions of false positives passing the two filters, and the total number of true positives. Given the number of genes (from the set of 2259) passing the first, the second, and the joint filters, we can write down three equations, and solve for the three unknowns (see methods). From the obtained parameters, we estimate that out of 2259 ORFs with CRCAAA motif in their promoters, 169 ± 20 are the regulatory targets of Ndt80. Among the 115 genes we identified as putative Ndt80 targets, 43 ± 5 are false positives. We also found that the probability for a functional MSE to be conserved across 4 species is 0.5 while the probability of chance conservation for a false positive site is 0.1. This gives a quantitative measure of the strength of selection on a functional regulatory site.

### Validating the predictions using gene expression profiles

**Figure 5:**
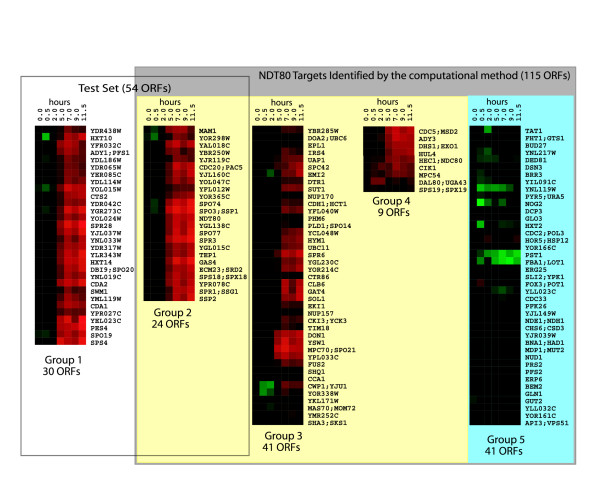
Clustering of the sporulation temporal expression profiles of genes in the test set and genes predicted by the computational method. The box on the left shows the profiles of genes in the test set (group 1 and 2). The 115 ORFs predicted by the computational method were shown in the right box (group 2 to 5). Group 2 is the overlap between the test set and the predicted set. The 91 ORFs in the predicted set only give rise to three distinct clusters (groups 3 – 5).

To validate these predictions, we performed a clustering analysis of the mRNA expression profiles during sporulation using the expression of the 115 genes predicted by our computational method and the 54 middle genes in the test set. We separately clustered the expression profiles of the following three sets of genes: 30 genes in the test set but missed by our computational analysis; 24 genes given by the overlap between the test set and the predicted set; and 91 genes in the predicted set but not in the test set. The first two sets of genes showed a clear middle gene expression profile – repressed till about 2 hours and then sharply turned on at 5 hours (group 1 and 2 in Figure 5). The set of 91 genes gives rise to three distinct clusters (group 3 – 5 in Figure 5). The first cluster (41 genes, group 3) resembles middle gene expression profile. The second cluster (group 4) contains 9 genes turned on at 0.5 hours and then at much higher level at 5 hours. The third cluster (group 5) contains 41 genes which are not up-regulated during sporulation. We believe that the 74 genes in groups 2–4 predicted by the computational method are likely to be the true targets of Ndt80, while the 41 genes in group 5 are likely false positives. This estimation of false positives is consistent with the number we obtained from the simple model (43 ± 5) presented above.

We also compared the targets predicted by our computational method to those identified by Ndt80 over-expression during vegetative growth [20]. We selected 236 genes whose log transformed expression ratio is 2 standard deviation above the mean as targets predicted by over-expression data. We found that 49 out of 54 genes in the test set, and 20 out of 50 in group 3 and 4 were predicted by Ndt80 over-expression. These overlaps were highly significant (only expect 2 by random for each case). For group 5 genes which we believe are false positives, the overlap is insignificant, only 2 out of 41 were predicted by the over-expression data.

Our predictions are also consistent with the microarray data for the Ndt80 deletion strain during sporulation[20]. We compared the fold changes for the mRNA induction of the Ndt80 deletion strain to the wild type strain. The fold change ratio is defined as r=(E6hrNdt80Δ/E2hrNdt80Δ)/(E7hrWT/E2hrWT)
 MathType@MTEF@5@5@+=feaafiart1ev1aaatCvAUfKttLearuWrP9MDH5MBPbIqV92AaeXatLxBI9gBaebbnrfifHhDYfgasaacH8akY=wiFfYdH8Gipec8Eeeu0xXdbba9frFj0=OqFfea0dXdd9vqai=hGuQ8kuc9pgc9s8qqaq=dirpe0xb9q8qiLsFr0=vr0=vr0dc8meaabaqaciGacaGaaeqabaqabeGadaaakeaacqWGYbGCcqGH9aqpcqGGOaakcqWGfbqrdaqhaaWcbaGaeGOnayJaemiAaGMaemOCaihabaGaemOta4KaemizaqMaemiDaqNaeGioaGJaeGimaaJaeuiLdqeaaOGaei4la8Iaemyrau0aa0baaSqaaiabikdaYiabdIgaOjabdkhaYbqaaiabd6eaojabdsgaKjabdsha0jabiIda4iabicdaWiabfs5aebaakiabcMcaPiabc+caViabcIcaOiabdweafnaaDaaaleaacqaI3aWncqWGObaAcqWGYbGCaeaacqWGxbWvcqWGubavaaGccqGGVaWlcqWGfbqrdaqhaaWcbaGaeGOmaiJaemiAaGMaemOCaihabaGaem4vaCLaemivaqfaaOGaeiykaKcaaa@5C93@, where we use the mRNA expression at 6 hours (E6hrNdt80Δ
 MathType@MTEF@5@5@+=feaafiart1ev1aaatCvAUfKttLearuWrP9MDH5MBPbIqV92AaeXatLxBI9gBaebbnrfifHhDYfgasaacH8akY=wiFfYdH8Gipec8Eeeu0xXdbba9frFj0=OqFfea0dXdd9vqai=hGuQ8kuc9pgc9s8qqaq=dirpe0xb9q8qiLsFr0=vr0=vr0dc8meaabaqaciGacaGaaeqabaqabeGadaaakeaacqWGfbqrdaqhaaWcbaGaeGOnayJaemiAaGMaemOCaihabaGaemOta4KaemizaqMaemiDaqNaeGioaGJaeGimaaJaeuiLdqeaaaaa@38E7@) and at 2 hours (E2hrNdt80Δ
 MathType@MTEF@5@5@+=feaafiart1ev1aaatCvAUfKttLearuWrP9MDH5MBPbIqV92AaeXatLxBI9gBaebbnrfifHhDYfgasaacH8akY=wiFfYdH8Gipec8Eeeu0xXdbba9frFj0=OqFfea0dXdd9vqai=hGuQ8kuc9pgc9s8qqaq=dirpe0xb9q8qiLsFr0=vr0=vr0dc8meaabaqaciGacaGaaeqabaqabeGadaaakeaacqWGfbqrdaqhaaWcbaGaeGOmaiJaemiAaGMaemOCaihabaGaemOta4KaemizaqMaemiDaqNaeGioaGJaeGimaaJaeuiLdqeaaaaa@38DF@) for the deletion strain and the mRNA expression at 7 hours (E7hrWT
 MathType@MTEF@5@5@+=feaafiart1ev1aaatCvAUfKttLearuWrP9MDH5MBPbIqV92AaeXatLxBI9gBaebbnrfifHhDYfgasaacH8akY=wiFfYdH8Gipec8Eeeu0xXdbba9frFj0=OqFfea0dXdd9vqai=hGuQ8kuc9pgc9s8qqaq=dirpe0xb9q8qiLsFr0=vr0=vr0dc8meaabaqaciGacaGaaeqabaqabeGadaaakeaacqWGfbqrdaqhaaWcbaGaeG4naCJaemiAaGMaemOCaihabaGaem4vaCLaemivaqfaaaaa@3418@) and 2 hours (E2hrWT
 MathType@MTEF@5@5@+=feaafiart1ev1aaatCvAUfKttLearuWrP9MDH5MBPbIqV92AaeXatLxBI9gBaebbnrfifHhDYfgasaacH8akY=wiFfYdH8Gipec8Eeeu0xXdbba9frFj0=OqFfea0dXdd9vqai=hGuQ8kuc9pgc9s8qqaq=dirpe0xb9q8qiLsFr0=vr0=vr0dc8meaabaqaciGacaGaaeqabaqabeGadaaakeaacqWGfbqrdaqhaaWcbaGaeGOmaiJaemiAaGMaemOCaihabaGaem4vaCLaemivaqfaaaaa@340E@) for the wild type strain. For Ndt80 targets, we expect that the fold induction during sporulation will be much less in the deletion strain compared to the wild type, thus *r *will be much smaller than 1, while for non-targets, we expect r to be around 1. The genome-wide average of the fold change ratio *r *is close to one (1.07 ± 0.57) and only 12% genes have the ratio less than 0.5. In group1, the fold change ratio of 29 genes out of 30 genes is less than 0.5 in the deletion strain. The fold change ratio of all genes in group 2 and 41 out of 50 genes in group 3 and 4 are less than 0.5, while only 8 out of 41 genes in group 5 have the ratio less than 0.5. The result for group 5 is consistent with the prediction that these genes are false positives based on the clustering analysis.

Examination of the promoter sequences of these genes reveals that different groups have the binding sites for other transcription factors in addition to the Ndt80 binding sites. For example, most genes in groups 2 and 3 (middle genes) have a binding site resembling that of both Ndt80 and Sum1 [29,31]. The binding of Sum1 may be important for repressing these genes early in sporulation. Two genes in group 4 (which are turned on early) *ADY3 *and *MPC54 *have the conserved URS1 site DSGGCGGC in their promoter sequences close to the conserved Ndt80 binding site. It is known that Ume6/Ime1 complex bind to URS1 and turn on their target genes early in sporulation[20,32]. Another gene with similar expression profile and conserved URS1 and Ndt80 sites is *YNL196C*. This gene is not in the cluster as the position of the Ndt80 site is -78, which barely missed our positional cutoff of -80. Thus it seemed that Ndt80 works together with other factors to fine tune the temporal expression of different gene sets in a combinatorial fashion.

### Comparison with ChIP-chip data

ChIP-chip technology has been widely used to identify the genomic targets of transcription factors. Recently, Harbison et al. published a comprehensive dataset for yeast containing 203 factors under a variety of conditions[2]. This dataset includes Ndt80 done under the YPD condition. We found that with the standard P value cut off (P < 0.001), 18 genes were predicted, and none is in the test set. With the less stringent cut off P < 0.01, 86 genes were predicted, and again none is in the test set. Only a few genes out of the 86 predicted by the ChIP data showed a middle gene expression profile (see additional file 1 and 4). Thus it seems that the ChIP-chip data for Ndt80 under YPD condition is dominated by noise. This is not surprising as the experiment was done under normal growth condition, and Ndt80 is activated only during sporulation. For Ndt80, the signal would be improved greatly by doing ChIP-chip experiment under sporulation condition. For an uncharacterized factor, it is quite challenging to search for conditions under which the factor is active. Although ChIP-chip is a powerful and systematic approach to dissecting transcription networks of a cell, the Ndt80 example highlighted the need for complementary approaches such as the one we described here.

## Discussion

The difficulty in successfully predicting regulatory elements and targets of transcription factors is exemplified by studies on meiotic middle genes activated via Ndt80 binding to the MSE. Although many middle genes have been identified from gene expression data and statistically significant over-representation of the MSE element in the promoters of these genes has been observed, predicting the target genes of Ndt80 with high sensitivity and specificity based on sequence information has been a big challenge. In fact, most consensus MSEs in the genome (>90%) are located upstream of genes not expressed during middle sporulation and not regulated by Ndt80. This problem is not Ndt80 specific – the appearance of a consensus-binding site upstream of a gene is neither necessary nor sufficient to indicate that the gene is regulated by a transcription factor known to bind to that site. Here we show that combining careful biochemical characterization of the binding site with positional information and evolutionary conservation in multiple species, we can significantly improve our ability to predict TF target genes. Using the combined information, we identified 115 putative targets of Ndt80 from about 2200 candidate genes whose promoter contains the consensus MSE. The predictions are quite specific (~40 false positives) and they include 45% of those from the positive test set. By combining our predictions based on sequence analysis with those from the test set selected based on both motif and gene expression data, we obtained 145 genes, with ~100 estimated to be true positives. We believe this set captured the majority of the Ndt80 targets (with the consensus MSE motif) in the genome, which is estimated to be ~170. These genes are good candidates for future experimental validation, and for analyzing combinatorial regulation by Ndt80 and other transcription factors.

The strength of this method is the application of many independent filters, each of which preferentially eliminated non-target genes. Based on quantitative analysis of relative binding affinity corroborated by *in vivo *experiments, we found that true positive sites have on average stronger binding than false positive sites (although they share the same consensus) due to the contributions from sequences flanking the consensus and from the degenerate positions. This allows us to eliminate about 50% false positives while keeping 80% of true positives. We have demonstrated by bioinformatic analysis as well as by experiments that the location of the MSE is important for middle gene regulation. This location preference is used to further eliminate false positives. When restricted to genes with MSE elements positioned from -80 to -400, we reduced the total number of predictions by a factor of 2 while keeping all the known targets in the test set. The conservation of the MSE across multiple yeast species is another powerful filter. By demanding conservation across 4 different yeast species, we reduced the total number of predictions by a factor of 10, while keeping half of the targets in the test set. The cross species comparison also provides insight into which MSE sequence is likely to be used when multiple MSE sequences are located in the same promoter. We have used conservation in a primitive way, by demanding exact conservation of the consensus sequence across all species. Targets were missed because the orthologous promoters do not exist in all species, the promoter sequences are seriously misaligned, or the binding site is not conserved at the consensus sequence level. We believe that the sensitivity of the search can be improved by using position specific weight matrix to quantitatively account for the variability of the sequence across species, and by using the phylogenetic tree to account for the evolutionary relationships.

A difficult task for genome-wide prediction is to estimate the false positive rate and the total number of targets, or equivalently, how many predicted targets are false positives and how many are missed. This is important for understanding the scope of the transcriptional response and for building confidence in the inferred transcriptional regulatory networks. For a factor with sufficient number of known targets, false negative rate can be estimated by the fraction of known targets missed. Estimating false positives is more difficult. Previously, false positive rates could be estimated only by experimentally verifying the predicted targets. Here we proposed a simple computational method to estimate false positive rate and the error of the estimation. For this method to work, one needs a set of high confidence targets and at least two independent filters to select potential targets. We presented the results where one filter is taken as positional distribution plus binding site score and another filter as conservation across 4 species. We have used a number of other combinations and the results are all consistent. Our estimation is also consistent with the results from the clustering analysis.

The approach we present is a general one that allows the identification of true targets of a transcription factor. There are a large number of transcription factors in the yeast genome for which a sufficient number of true targets are known and PSWMs are available. Our approach can be used to make better predictions of the targets for these factors and to estimate false positive and false negative rates. For uncharacterized transcription factors, our study suggests that in vitro characterization of the binding specificity (e.g., systematic data from SELEX experiments) followed by a genomic search using positional constraint and evolutionary conservation can be an efficient way for identifying their regulatory targets. This approach will complement other genomic approaches that determine the regulatory targets of a transcription factor.

## Methods

### Molecular biology and strains

MBP-Ndt80 fusion constructs and their purification, EMSA *in vitro *binding analysis, Northern blot analysis and all *in vivo *gene and element replacements were done as a variation of previously described techniques and are further described in supplementary materials (Additional file 1). β-galactosidase experiments were done in W303 *MAT***a ***ade2-1 trp1-1 can1-100 leu2-3,12 ura3-52 his3-11,15*; all other experiments were done in yeast strain background SK1 *MAT***a**, *MATα*, or **a**/α *ho::his G ura3 lys2 leu2::his G trp*Δ*FA his3-11,15*. Strain yEJ129 is *MAT***a **Pspo77 *mse::URA3 *where the MSE sequence at the *SPO77 *promoter is replaced with *URA3*; strain yEJ152 is yEJ129 with *GFP*-*TRP1 *inserted into the *SPO77 *locus.

### Sequence data

We download the sequences for *S. cerevisiae*, *S. paradoxus*, *S. mikatae*, and *S. bayanus *published by Kellis et al [11]. The sequences files contain 5306 *S. cerevisiae *ORFs and their promoters and the corresponding orthologous sequences in other species.

### Binding score

To calculate the binding score for a potential site, we define position specific scoring matrix Eit=ΔGWT−ΔGMitkT
 MathType@MTEF@5@5@+=feaafiart1ev1aaatCvAUfKttLearuWrP9MDH5MBPbIqV92AaeXatLxBI9gBaebbnrfifHhDYfgasaacH8akY=wiFfYdH8Gipec8Eeeu0xXdbba9frFj0=OqFfea0dXdd9vqai=hGuQ8kuc9pgc9s8qqaq=dirpe0xb9q8qiLsFr0=vr0=vr0dc8meaabaqaciGacaGaaeqabaqabeGadaaakeaacqWGfbqrdaqhaaWcbaGaemyAaKgabaGaemiDaqhaaOGaeyypa0ZaaSaaaeaacqqHuoarcqWGhbWrdaahaaWcbeqaaiabdEfaxjabdsfaubaakiabgkHiTiabfs5aejabdEeahnaaCaaaleqabaGaemyta00aa0baaWqaaiabdMgaPbqaaiabdsha0baaaaaakeaacqWGRbWAcqWGubavaaaaaa@4144@, where Δ*G *measures the standard free energy change of protein-DNA complex formation, *WT *stands for the wild type DNA sequence, and Mit
 MathType@MTEF@5@5@+=feaafiart1ev1aaatCvAUfKttLearuWrP9MDH5MBPbIqV92AaeXatLxBI9gBaebbnrfifHhDYfgasaacH8akY=wiFfYdH8Gipec8Eeeu0xXdbba9frFj0=OqFfea0dXdd9vqai=hGuQ8kuc9pgc9s8qqaq=dirpe0xb9q8qiLsFr0=vr0=vr0dc8meaabaqaciGacaGaaeqabaqabeGadaaakeaacqWGnbqtdaqhaaWcbaGaemyAaKgabaGaemiDaqhaaaaa@30CA@ stands for a mutant with a single base mutation *t *at position *i*. The binding score for an arbitrary motif is defined as the summation of the position specific scores B=∑i=1NEiti
 MathType@MTEF@5@5@+=feaafiart1ev1aaatCvAUfKttLearuWrP9MDH5MBPbIqV92AaeXatLxBI9gBaebbnrfifHhDYfgasaacH8akY=wiFfYdH8Gipec8Eeeu0xXdbba9frFj0=OqFfea0dXdd9vqai=hGuQ8kuc9pgc9s8qqaq=dirpe0xb9q8qiLsFr0=vr0=vr0dc8meaabaqaciGacaGaaeqabaqabeGadaaakeaacqWGcbGqcqGH9aqpdaaeWbqaaiabdweafnaaDaaaleaacqWGPbqAaeaacqWG0baDdaWgaaadbaGaemyAaKgabeaaaaaaleaacqWGPbqAcqGH9aqpcqaIXaqmaeaacqWGobGta0GaeyyeIuoaaaa@3B0D@, where *t*_*i *_is the nucleotide type of the motif at position *i*. The standard free energy change is calculated from the fraction of DNA bound based on the law of mass action (see the supplementary materials in Additional file 1 for details).

### Cross species analysis

We search for the core motif CRCAAA in the promoter regions of the four yeast species (*S. cerevisiae*, *S. bayanus*, *S. paradoxus*, and *S. mikatae*) in a positional window *x *± 20 relative to their respective translation start sites and count the number of species in which the core motif was found in the window. We maximize this number with respect to *x *and define it as the number of species in which the motif is conserved. This definition allows us to identify conserved core motifs even if the promoter sequences are slightly misaligned.

### Estimation of the total number of targets and the false positive rate

To estimate the false positive rate of our prediction and the total number of targets in the genome, we screened the 2259 ORFs whose promoter contains the consensus CRCAAA motif by two different filters. The first filter selects ORFs whose binding affinity score is larger than -0.4 and the location is between -80 and -400 bps upstream. The second filter selects ORFs in which the consensus sequence is conserved across all 4 species. We applied the two filters separately and jointly to the set of 2259 ORFs and recorded the number of ORFs passing the first, second, and both filters as 573, 294, and 115 respectively. Using x to denote the number of true targets among the 2259 ORFs, we derived the following equations:

α_1_*x *+ β_1_(2259 - *x*) = 573

α_2_*x *+ β_2_(2259 - *x*) = 294

α_1_α_2_*x *+ β_1_β_2_(2259 - *x*) = 115

where α_1_, α_2 _are the fractions of the true targets passing the first filter and the second filter, and β_1_, β_2 _are the fractions of the false positives passing the first and the second filter. We made the assumption that the two filters are independent of each other, so that the fraction of true and false positives passing both filters can be written as a product of the fractions passing each individual filter. We found that this assumption is quite good for the true positives. For false positives, we cannot directly assess how good the assumption is. We argue that since the conservation of a false positive site is by chance, it is unlikely to correlate with the binding site affinity score and the location. To estimate α's, we applied the some filters to 54 ORFs from the test set of true positives and recorded the number of ORFs passing the first, the second, and both filters as 46, 27, and 24, from which we obtained α_1 _= 46/54 = 0.85, α_2 _= 27/54 = 0.50. The independence assumption predicts that the fraction passing both filters is α_1_α_2 _= 0.43, which agrees well with the observed value 24/54 = 0.44.

Given α's, we solved the above equations to obtain β_1 _= 0.21, β_2 _= 0.10, and *x *= 169, which gives the total number of true targets in the set of 2259 ORFs. From these numbers we obtained the number of false positive predictions in our final 115 ORF list is 43. As a by-product, the probability by chance a false positive CRCAAA motif is conserved across 4 species is estimated to be 0.10. We used bootstrap to estimate the errors. Using the above parameters, we sample the number of ORFs passing the first, second, and both filters using binomial distribution, and re-calculated the parameters to get the standard deviations. We found that δ*x *= 20, δβ_1 _= 0.02, δβ_2 _= 0.01, δα_1 _= 0.03, δα_2 _= 0.04, and the standard deviation for the false positives is 5.

## Authors' contributions

E. J. and I. H. conceived and designed the experiments. E. J. performed the experiments. C-S. C. and H. L. conceived and designed the computational analysis. C-S. C. carried out the computational analysis. E.J., C.-S. C., and H. L. wrote the paper.

## Supplementary Material

Additional File 1 Supplemental Materials. Click here for file

Additional File 2 Quantitative analysis of *in vivo *expression driven by MSE variants. Northern hybridization bands were analyzed on phosphoImager. *SPO77 *and *GFP *levels are quantitated relative to the loading control *PFY1*. Click here for file

Additional File 3 The location of the MSE is critical for sporulation specific expression of RNA at the *SPO77 *locus. In a heterologous strain where *SPO77 *is replaced with *GFP *at one locus, the MSE was relocated to positions (a) -450 or (c) -50 and the endogenous MSE at -152 was mutated to a non-functional MSE. As a control, the MSE was inserted at positions (b) -450 and (d) -50 in a strain where the endogenous MSE is functional and unchanged. Click here for file

Additional File 4 Clustering diagram of microarray expression[1] for those genes identified as NDT80 targets by the ChIP-on-Chip experiments[9] using p-value < 0.01. Click here for file
