# Supplementary material for: Genome-wide identification of the regulatory targets of a transcription factor using biochemical characterization and computational genomic analysis
Source: BMC Bioinformatics. 2005 Nov 18;6:275. doi: 10.1186/1471-2105-6-275 (PMC1326232; doi:10.1186/1471-2105-6-275)
Supplement: Additional File 1 — Supplemental Materials. [file 1471-2105-6-275-S1.doc]

# SUPPLEMENTARY MATERIALS

# MBP-Ndt80 fusion constructs. MBP-Ndt80, was made by cloning the Bgl II/ Sal I Ndt80-containing fragment of pSC086 [1] into the BamH I/ Sal I site of vector pMAL-c2 (New England Biolabs) to make plasmid pEJ145. MBP-Ndt80, 51-350 was made by cloning the ~900bp BamHI/ BglII fragment of Ndt80 from pEJ099 into the BamHI site of pMAL-c2 to make plasmid pEJ151.

# Purification of MBP-Ndt80p fusions. MBP-Ndt80 fusion proteins were expressed in BL21 (DE3 Codon Plus) bacterial cells. At OD_600_ 0.5, 100mL of cells were induced for 2 hours with IPTG to final concentration of 0.4mM. Cell pellets were lysed by sonication in phosphate lysis buffer (5% 1M Phosphate Buffer, pH 8.0, 200mM NaCl, 0.1 mg/mL lysozyme, 0.34 mg/mL PMSF, EDTA free protease cocktail [Roche]. The lysate was cleared by centrifugation 14K for 30 minutes at 4 °C and purified in batch with 500 μL amylose beads (New England Biolabs).

# EMSA *in vitro* binding analysis. *In vitro* binding assays were performed as follows. One microgram of purified MBP-Ndt80 protein or purified MPB-Ndt80, 51-350 was incubated with unlabeled competitor probe for 30 min prior to addition of 100 fmol of ^32^P labeled DNA in 20 μL of 1X Buffer M (Boehringer). Reaction were incubated for 20 minutes at room temperature. Double-stranded DNA probes were made by annealing 35 base-pair DNA oligos slowly from 99 °C to 4 °C leaving 5’ GG overhangs in STE buffer as described previously [2]. DNA probes were labeled using the Klenow fragment of DNA polymerase (Boehringer Mannheim) and (α-^32^P]dCTP. Protein-DNA reaction mixtures were loaded onto a 5% nondenaturing polyacrylamide gel and electrophoresed at 200V in 0.5X Tris-borate-EDTA buffer at 4 °C. The gel was dried and exposed to film or analyzed and quantitated with a PhosphoImager (Molecular Dynamics).

**Northern blot analysis**. RNA was harvested from synchronized sporulating cells. Northern analysis was done using commercially available Rapid Hybe Buffer (Amersham Biosciences) as directed, with Prime-It Kit (Stratagene) random-prime labeled probes. All probes were PCR amplified from genomic sequence and were gel purified. The blots were stripped with 50% formamide, reprobed sequentially, and analyzed with a PhosphoImager (Molecular Dynamics).

**Yeast strains**. All beta-galactosidase experiments were done in yeast strain background W303 *MAT***a** *ade2-1 trp1-1 can1-100 leu2-3,12 ura3-52 his3-11,15*. All other experiments were done in yeast strain background SK1 *MAT***a**, *MATα,* or **a**/α *ho::his G ura3 lys2 leu2::his G trpΔFA his3-11,15*. Strain yEJ129 is *MAT***a** Pspo77 *mse::URA3* where the MSE sequence at the *SPO77* promoter is replaced with *URA3* by one-step replacement using Cg*URA3* PCR product [3]. Insertion was tested by PCR. *GFP*-*TRP1* was inserted into the *SPO77* locus of yEJ129 by one-step recombination using *GFP* and *TRP1* amplified by PCR from plasmid pFA6a-*GFP*(S65T)-*TRP1* to make strain yEJ152 and tested by PCR [4].

All *in vivo* gene and element replacements were done as a variation of previously described techniques [5]. To make *in vivo* MSE variants at the *SPO77* locus, strain yEJ152 was transformed with various plasmids fragments containing a 710 base-pair *SPO77* promoter containing region with either no change or a single base-pair change to the MSE sequence.

**Mutagenesis of the MSE at *SPO77*.** The promoter region of *SPO77* was amplified by PCR from -710 to -1 nucleotides relative to the translation start site. The 5’ oligo is located 150 nucleotides in the *RPP0* locus, an essential gene encoding a cytoplasmic component of the ribosome [6]. The 710 base-pair PCR construct containing the *SPO77* promoter was cloned into the pCR2.1 TA cloning vector (Invitrogen) to make plasmid pEJ212, which was subsequently sequenced. Plasmid pEJ212 was subjected to QuickChange site-directed mutagenesis as described by Stratagene and transformed into DH5α competent bacterial cells. All mutants were sequenced to ensure proper single base-pair changes. Plasmids with various MSE sequences at the *SPO77* promoter were then digested with EcoRI to release the *SPO77* promoter containing the MSE variant of interest and transformed into yeast strain yEJ152, replacing *URA3* by one step replacement and tested by sequencing. All of these *MAT***a** haploid strains were mated to WT SK1 *MAT*α cells to make the heterologous diploid strain.

**MSE Location Variants**. Quick-Change site directed mutagenesis was used to insert a MSE at either positions –50 or –450 of *the SPO77* promoter in plasmids pEJ212 and pEJ220. Briefly, oligonucleotide pairs oEJ230 and 231 were used for insertion of an MSE at position –450 from the translation start site in the *SPO77* promoter in plasmids pEJ212 and pEJ220 to make plasmids pEJ240 and pEJ230 respectively. Likewise, oligonucleotide pairs oEJ234 and oEJ235 were used for insertion of an MSE at position –50 in plasmids pEJ212 and pEJ220 to make plasmids pEJ235 and pEJ233, respectively.

MSE location variants strains were made by replacing nucleotides –300 through –450 in the *SPO77* promoter of yEJ170 with the URA3 gene. The *URA3* gene was subsequently replaced with EcoRI digested MSE location variant plasmids pEJ240, pEJ230, pEJ235, and pEJ233 to make yeast strain yEJ300, yEJ292, yEJ294, and yEJ295 respectively. These strains were subsequently mated with SK1*MAT*α WT cells to make the diploid heterozygous mutant.

**Sequence Data**. We download the gene sequences for *S. cerevisiae*, *S. paradoxus*, *S. mikatae*, and *S. bayanus* published by Kellis et al [7] (<http://www.broad.mit.edu/annotation/fungi/ comp_yeasts/>). The sequences files contain 5306 *S. cerevisiae* ORFs and the sequence of some of the corresponding orthologs in the other species. Since the authors use a more stringent definition for the ORFs, some of the earlier ORFs used the microarray experiments done by Chu et al [8] are not included in the sequence files. In the set of the middle genes identified by using the expression data at 2 and 5 hours, the following ORFs are not included in the *S. cerevisiae* sequence file: *YDL187C, YDR522C,* *YDR523C, YER180C, YER182W, YHR015W, YIL099W, YLR213C, YNL205C, YNL319W, YPR077C*.

**Normalization of the positional distribution of the core motif**. The normalized count for the positional distribution is defined as, where $C^{i}$ is the number of motifs in *i*th bin, is the number of promoter sequences available for the position used for *i*th bin, and $S_{\text{total}}$ is the total number of promoter sequences. For example, for the bin position at –325 to –350, there are only 3451 ORF sequences with upstream sequences longer than 350, out of a total of 5281 ORF sequences in the *S. cerevisiae* sequence file. If there are 101 CRCAAA motifs in this bin in the genome, then the normalized count is . This normalization procedure allows us to correct the bias of the position distribution due to the different length of the promoter region in the sequence files.

**Binding Score**. We define the binding score for a motif as the predicted difference of the binding free energies for the motif of interest and the wild type *SPO77* MSE. Consider the chemical reaction, P + DNA ↔ P|DNA, where P denotes the transcription factor, DNA represents the DNA oligo and P|DNA stands for the protein-DNA complex. The law of mass action relates the equilibrium concentrations to the standard free energy change. For the wild-type SPO77 MSE, we have

.

where$u$ is a molecular volume factor, and ${DG}^{\text{WT}}$ is the stand free energy change upon binding to DNA. Similarly, we have the following equation for the binding to a mutant version of MSE,

Under the experimental conditions, ; thus we can approximate the free protein concentration by the total protein concentration, and use the following equation to determine the difference of the standard free energy change between the wild type and the mutant, , where the ratio is determined from the gel shift experiments. We define position specific scores , where $M_{i}^{t}$ is a mutant with a single base mutation $t$ at position $i$. The binding score for an arbitrary motif is defined as the summation of position specific scores , where $t_{i}$ is the nucleotide type of the motif at position $i$. By definition, the wild type *SPO77* MSE has a score $B=0$. MSE sequences with stronger binding than the wild type sequence have binding scores larger than 0.

**SUPLEMENTARY FIGURE LEGEND**

Figure S1. Quantitative analysis of *in vivo* expression driven by MSE variants. Northern hybridization bands were analyzed on phosphoImager. *SPO77* and *GFP* levels are quantitated relative to the loading control *PFY1*.

Figure S2. The location of the MSE is critical for sporulation specific expression of RNA at the *SPO77* locus. In a heterologous strain where *SPO77* is replaced with *GFP* at one locus, the MSE was relocated to positions (a) –450 or (c) –50 and the endogenous MSE at –152 was mutated to a non-functional MSE. As a control, the MSE was inserted at positions (b) –450 and (d) –50 in a strain where the endogenous MSE is functional and unchanged.

Figure S3. Clustering diagram of microarray expression[1] for those genes identified as NDT80 targets by the ChIP-on-Chip experiments[9] using p-value < 0.01.

1. Chu S, Herskowitz I: **Gametogenesis in yeast is regulated by a transcriptional cascade dependent on Ndt80**. *Mol Cell* 1998, **1**(5):685-696.

2. Sgarbanti M, Borsetti A, Moscufo N, Bellocchi MC, Ridolfi B, Nappi F, Marsili G, Marziali G, Coccia EM, Ensoli B *et al*: **Modulation of human immunodeficiency virus 1 replication by interferon regulatory factors**. *J Exp Med* 2002, **195**(10):1359-1370.

3. Kitada K, Yamaguchi E, Arisawa M: **Cloning of the Candida glabrata TRP1 and HIS3 genes, and construction of their disruptant strains by sequential integrative transformation**. *Gene* 1995, **165**(2):203-206.

4. Longtine MS, McKenzie A, 3rd, Demarini DJ, Shah NG, Wach A, Brachat A, Philippsen P, Pringle JR: **Additional modules for versatile and economical PCR-based gene deletion and modification in Saccharomyces cerevisiae**. *Yeast* 1998, **14**(10):953-961.

5. Guthrie C, Fink G: **Methods in Enzymology: Guide to Yeast Genetics and Molecular Biology**, vol. 194; 1991.

6. Santos C, Ballesta JP: **Ribosomal protein P0, contrary to phosphoproteins P1 and P2, is required for ribosome activity and Saccharomyces cerevisiae viability**. *J Biol Chem* 1994, **269**(22):15689-15696.

7. Kellis M, Patterson N, Endrizzi M, Birren B, Lander ES: **Sequencing and comparison of yeast species to identify genes and regulatory elements**. *Nature* 2003, **423**(6937):241-254.

8. Chu S, DeRisi J, Eisen M, Mulholland J, Botstein D, Brown PO, Herskowitz I: **The transcriptional program of sporulation in budding yeast**. *Science* 1998, **282**(5389):699-705.

9. Harbison CT, Gordon DB, Lee TI, Rinaldi NJ, Macisaac KD, Danford TW, Hannett NM, Tagne JB, Reynolds DB, Yoo J *et al*: **Transcriptional regulatory code of a eukaryotic genome**. *Nature* 2004, **431**(7004):99-104.
