# Supplementary figures and images for: Genome-wide identification of the regulatory targets of a transcription factor using biochemical characterization and computational genomic analysis
Source: BMC Bioinformatics. 2005 Nov 18;6:275. doi: 10.1186/1471-2105-6-275 (PMC1326232; doi:10.1186/1471-2105-6-275)

Figure S1

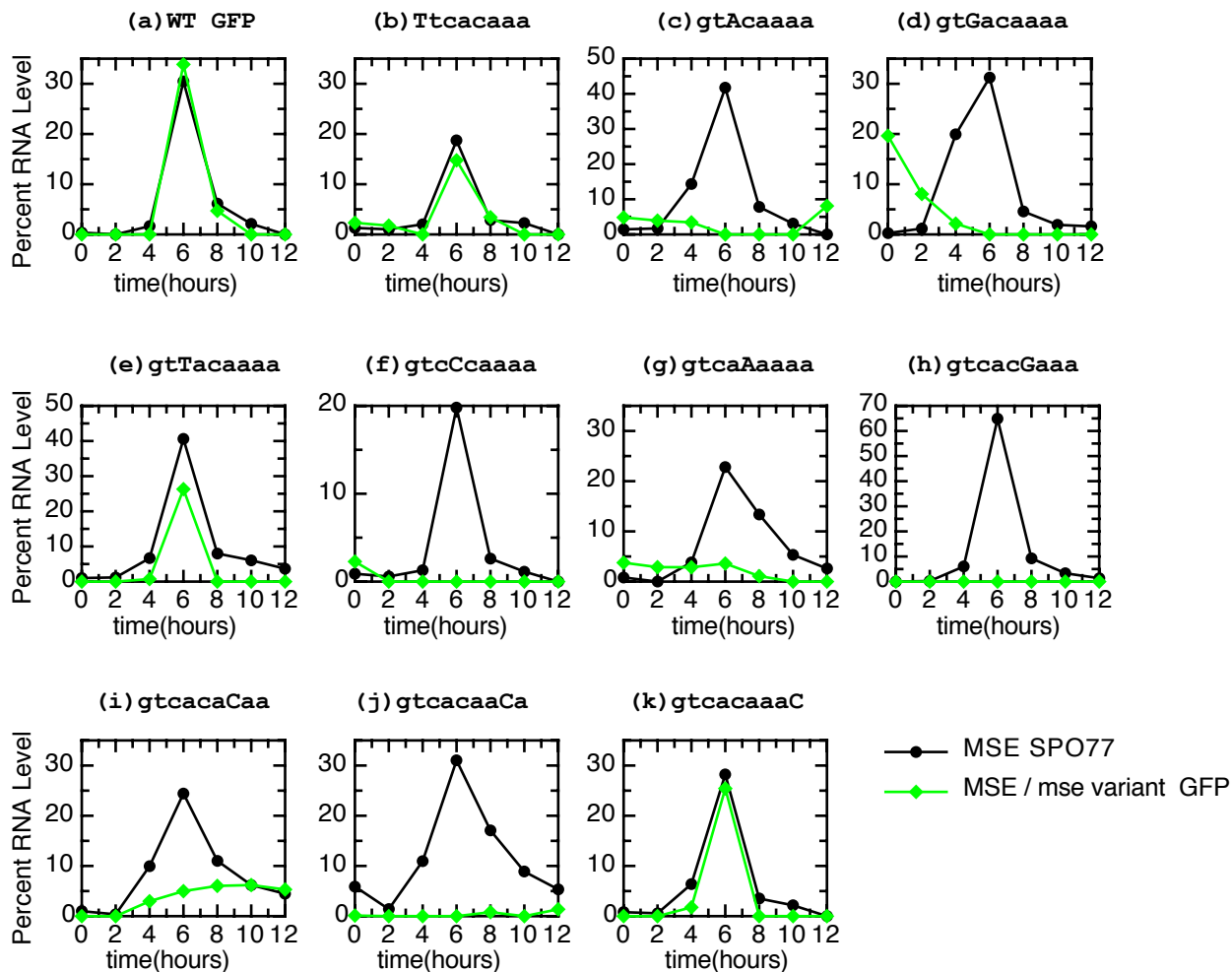

Supplement: Additional File 2 — Quantitative analysis of in vivo expression driven by MSE variants. Northern hybridization bands were analyzed on phosphoImager. SPO77 and GFP levels are quantitated relative to the loading control PFY1. [file 1471-2105-6-275-S2.pdf]

Figure S2

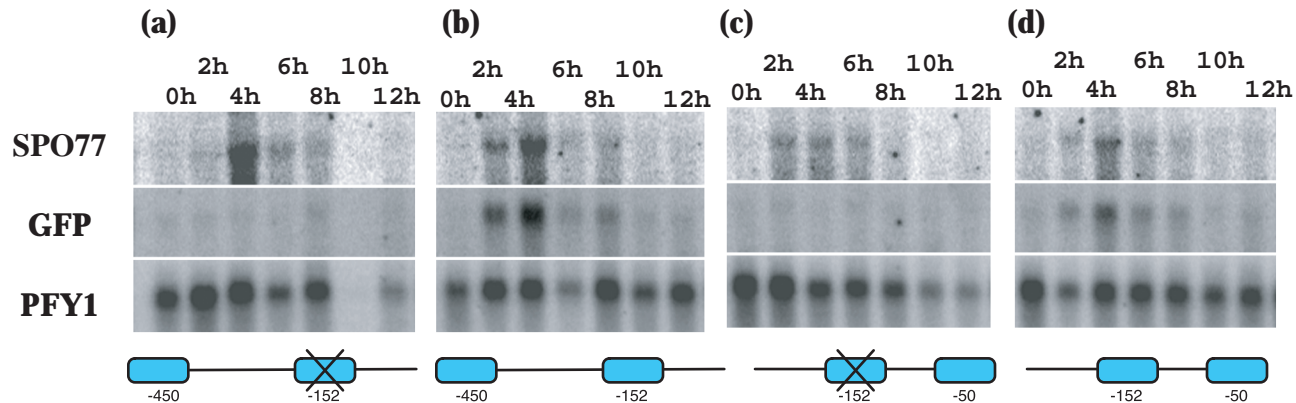

Supplement: Additional File 3 — The location of the MSE is critical for sporulation specific expression of RNA at the SPO77 locus. In a heterologous strain where SPO77 is replaced with GFP at one locus, the MSE was relocated to positions (a) -450 or (c) -50 and the endogenous MSE at -152 was mutated to a non-functional MSE. As a control, the MSE was inserted at positions (b) -450 and (d) -50 in a strain where the endogenous MSE is functional and unchanged. [file 1471-2105-6-275-S3.pdf]

Figure S3

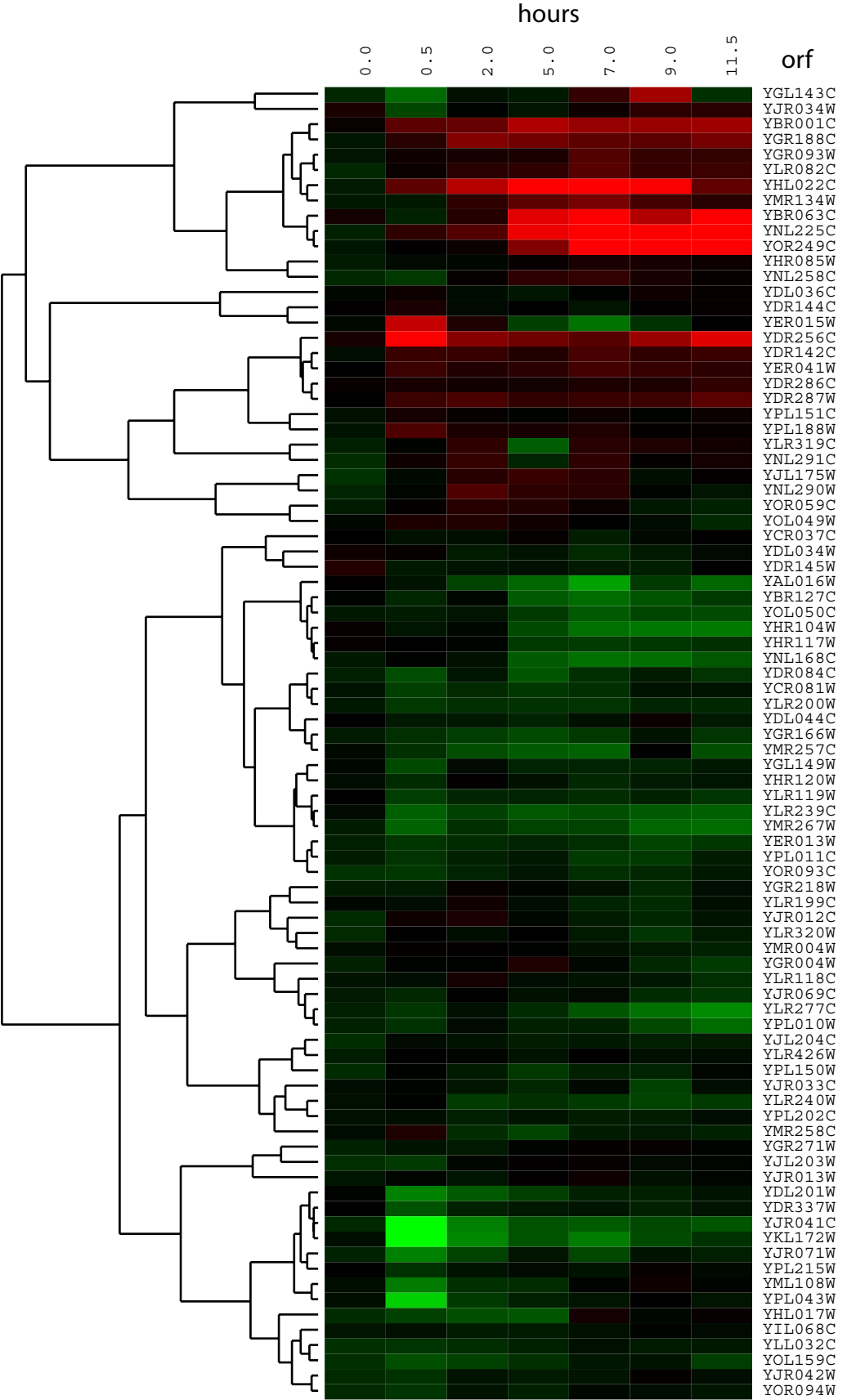

Supplement: Additional File 4 — Clustering diagram of microarray expression[1] for those genes identified as NDT80 targets by the ChIP-on-Chip experiments[9] using p-value < 0.01. [file 1471-2105-6-275-S4.pdf]
